# Prevalence of mesiobuccal-2 canals in maxillary first and second molars among the Bruneian population—CBCT analysis

**DOI:** 10.1038/s41405-022-00125-5

**Published:** 2022-11-19

**Authors:** Hui Yi Onn, Malissa Siao Yun Abdullah Sikun, Hanif Abdul Rahman, Jagjit Singh Dhaliwal

**Affiliations:** 1 PAPRSB Institute of Health Sciences, Universiti Brunei Darussalam, Gadong, Bandar Seri Begawan, BE1410 Brunei Darussalam; 2 Ministry of Health, Department of Dental Services, Bandar Seri Begawan, Brunei Darussalam

**Keywords:** Health care, Endodontics

## Abstract

**Introduction:**

Identification of the second mesiobuccal canal (MB-2) in maxillary molars is considered an endodontic concern of many practitioners due to its complex morphology. The use of Cone-beam Computed Tomography (CBCT) is a necessity for easier location of this elusive canal during endodontic treatment.

**Aim:**

To study the prevalence of the MB-2 canal in the maxillary first and second molars amongst the Bruneian population.

**Materials and methods:**

A retrospective study involving a review of scans taken from a CBCT scanner (J Morita; Veraviewepocs 3D R100 Panoramic/Cephalometric) over a 5-year period, from May 2016 to May 2021 was carried out. A total of 342 maxillary molars were evaluated independently by two observers. Any contradicting outcomes were discussed by both observers until a consensus was reached. In addition, the correlation of MB-2 canals with gender and age were calculated using the chi-squared test.

**Results:**

The prevalence of MB-2 canal in the maxillary first and second molars are 51.3% and 29.8% respectively. Both males and females have a similar prevalence of MB-2 canals in the maxillary first and second molars. The incidence of MB-2 canals in both maxillary first and second molars significantly decreases with increasing age. No significant correlation between the prevalence of MB-2 canals with different gender groups in the population.

**Conclusions:**

It is crucial for clinicians to identify the presence of MB-2 canals when performing endodontic treatment of the maxillary first and second molars. Varying prevalence has been reported for different populations. Recognising this wide-ranging prevalence amongst different populations will allow for greater predictability in ensuring endodontic treatment success.

## Introduction

Root canal treatment carries a success rate of up to 99.4% in both vital and non-vital teeth [1–3]. However, when treatment fails, clinicians are often confronted with challenges to improve the outcome of subsequent retreatment. Identification of the cause(s) of failed root canal treatment is crucial for the success of any endodontic intervention. One of the potential sources of persistent endodontic infection, particularly in maxillary first and second molars, is failure to locate and treat the entire root canal system during primary root canal treatment [4]. Canals can be missed and this is frequently seen in the mesiobuccal (MB) root of maxillary molars [5–8]. The MB root canal can be divided into two canals, namely, the first (MB-1) and second (MB-2) canals The morphology of the MB-2 canal with its mesiopalatal inclination to the orifice, makes it difficult to locate and negotiate during endodontic treatment [9, 10]. Moreover, diagnostic tools such as the two-dimensional conventional periapical radiographs offer little to improve the location of this elusive canal. With the help of an imaging modality with high precision such as the cone-beam computed tomography (CBCT), locating MB-2 canals in maxillary molars prior to endodontic treatment has been made possible [11–16].

Research has been conducted to identify the prevalence of the MB-2 canals in maxillary first and second molars using CBCT scans across many countries in Asia, specifically India, Malaysia, Thailand, Taiwan, and South Korea. The prevalence of MB-2 canals in maxillary first molars ranges from 36.3% to 86.8%, whereas maxillary second molars range from 8.5 to 82.6% [14, 15, 17–21]. Different demographic factors such as age, gender, and ethnicity may contribute to the varying prevalences of the MB-2 canal in the maxillary first and second molars.

Currently, there is no published data available on the prevalence of MB-2 canals in the Bruneian population. This study aims to provide such data to equip our dental practitioners with additional knowledge that may be beneficial whilst performing root canal treatment on maxillary molars. Though there are variations of the root anatomy of the MB-2 canal, it is not within the scope of this research. However, identification of the MB-2 canals merging to or diverting from the main canal allows better treatment outcomes of root canal treatment. In addition, the data obtained will contribute to the currently available evidence in the literature from other parts of the world.

## Aim

To study the prevalence of the MB-2 canal in the maxillary first and second molars in the Bruneian population.

## Objectives

1. To determine the prevalence of MB-2 canals in maxillary first and second molars,
2. To observe the associations between the presence of MB-2 canals in maxillary first and second molars and specific demographic factors.

## Materials and methods

### Study design

This was a retrospective study involving a CBCT database collected over a period of 5 years, from May 2016 to May 2021 in Brunei Darussalam.

### Methods

**Fig. 1:**
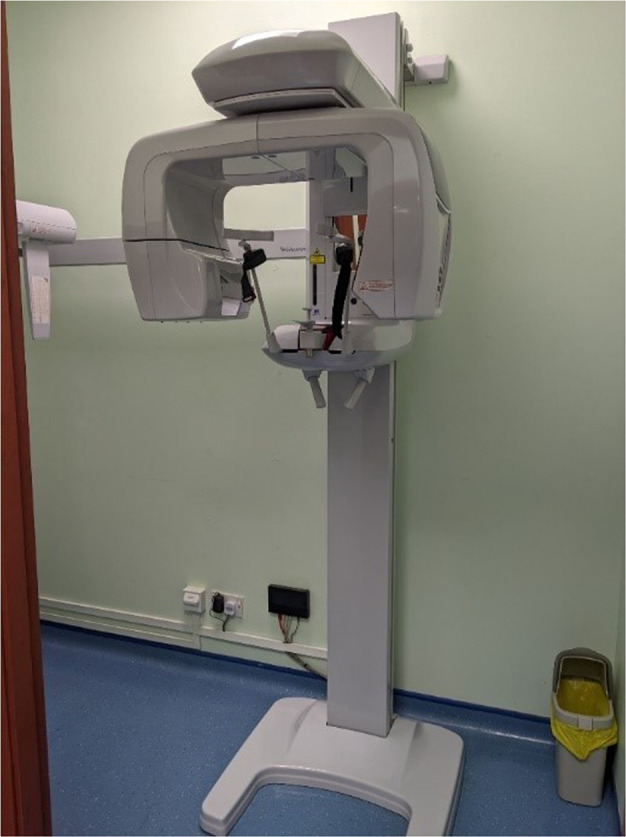
CBCT scanner. CBCT scanner used in the study which is located in the National Dental Centre, Brunei Darussalam.

**Fig. 2:**
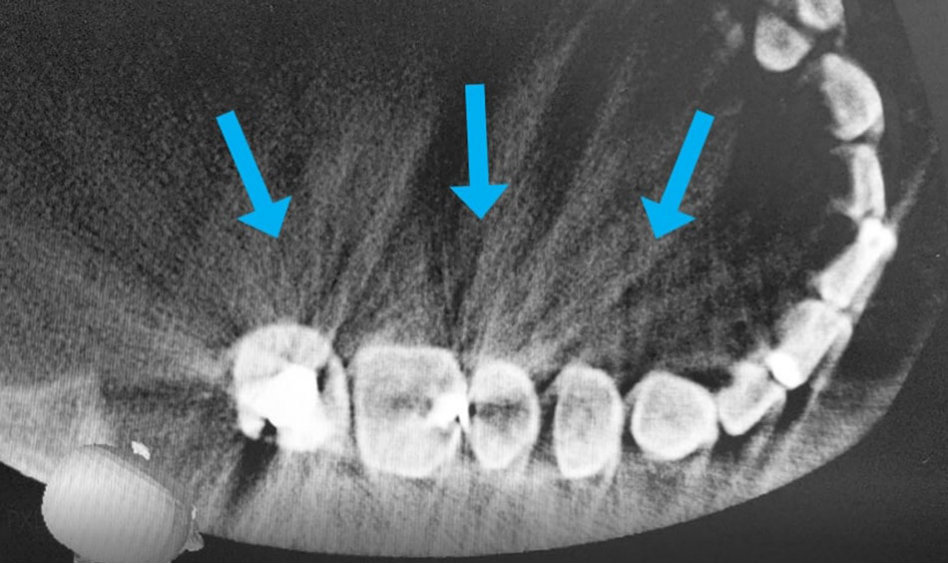
CBCT scan of teeth with amalgam restorations. Hyperdense streaks exhibited on the teeth with amalgam restorations on a CBCT scan.

Prior to commencing the study, ethical approval was obtained from the Joint Institute of Health Sciences of Universiti Brunei Darussalam Research Ethics Committee and the Ministry of Health Research Ethics Committee (Ethics reference number: UBD/PAPRSBIHSREC/2021/48). All requests for CBCT scans of the maxillary molar region taken during the stipulated time frame were collected. A total of 460 requests were identified and extracted. These scans were performed by a single CBCT machine (Morita Veraviewepocs 3D R100 Panoramic/Cephalometric, Kyoto, Japan) and operated throughout with an exposure setting of 60–90 kV,1–10 mA and an exposure time of about 9.4 s (Fig. 1). The field of view was kept to as low as reasonably practicable with either the Ø40mm × H40mm or Ø80mm × H40mm being employed depending on the requested number of teeth to be scanned in the quadrant or arch. The scans were then reconstructed using the iDixel application software (Morita, Kyoto, Japan) (Fig. 1). The data collection was carried out by two examiners namely the principal researcher and a specialist endodontist (second author). All 460 CBCT scans were viewed and examined by both examiners. A positive identification of the presence or absence of the MB-2 canal must be obtained by both examiners. Any inconclusive findings were discussed and concurrently evaluated until a consensus is reached. In addition, any identified MB-2 canal was further examined and its morphology in relation to its confluence to the MB-1 canal as a single apical foramen/exit or a distinctly separate canal as two apical foramina/exits at the apical third of the root was recorded. All data were recorded in a Microsoft Excel spreadsheet before analysis. The following criteria was employed:

- Inclusion criteria:
  1. MB root of maxillary first and second molars
  2. Both genders
  3. Age 16 years and above
  4. Patients that possess a Brunei National Identification Card Type Yellow (Citizen of Brunei Darussalam) and Type Purple (Permanent Resident of Brunei Darussalam)
- Exclusion criteria:
  1. CBCT scans that without maxillary first and second molars
  2. Patients that possess a Brunei National Identification Card Type Green (Temporary Resident of Brunei Darussalam)
  3. CBCT scans with exhibition of hyperdense streaks (Fig. 2)

Nationality of the patients was obtained from mandatory demographic information written onto the CBCT scan request forms. Hyperdense streaks observed were generated from beam scattering in the presence of metallic structures in the oral cavity such as amalgam restorations, orthodontic brackets, or crowns (Fig. 2). These streaks could potentially disrupt the examination of the CBCT scans and give an inaccurate observation. Therefore, CBCT scans with these streaks were omitted from the data.

### Statistical methods

A minimum sample size of 210 was required to achieve precision (power) of 5% (d = 0.05) in a study population of 460 CBCT scans at 50% prevalence rate and at 95% confidence interval [22]. Descriptive statistics were used to describe the sample characteristics consisting of the sample age and gender. Approximation method using 95% confidence interval was computed to calculate the prevalence of MB2 canal and the number of apical exits in maxillary first and second molars. Gender- and age- stratified associations of the proportion of MB-2 cases were computed using chi-square test for independence. The data were entered and analysed using RStudio Desktop software version 1.4.1106. In all statistical tests, the *p* value < 0.05 was considered statistically significant.

## Results

Out of the 460 CBCT scans examined, a total of 263 scans fulfilled the inclusion criteria and were analysed in this study. A total of 337 maxillary molars, consisting of 156 first molars and 181 second molars, were observed in the 263 scans. Table 1 shows the prevalence of these cases. The overall prevalence of MB-2 in the maxillary first molar (51.3%, 95% CI: 51.3, 59.3) was higher than maxillary second molar (29.8%, 95% CI: 23.4, 37.1). Those aged between 16 and 50 years old had shown a higher prevalence of MB-2 canals in both molars than ages above 50 years old. Among the CBCT scans observed, there was a higher percentage of MB-2 canals in the maxillary first molars among females (53.7%, 95% CI: 43.2, 63.9) than males (47.5%, 95% CI: 34.8, 60.6). However, the frequency distribution was similar for the maxillary second molars for females (27.4%, 95% CI: 19.7, 36.8) and males (33.8%, 95% CI: 23.1, 46.4).

**Table 1:** The prevalence of MB-2 canals in maxillary first and second molars by overall, gender and age groups (*n* = 337).

	95% Confidence interval			
	Frequency (n)	Percentage (%)	Lower limit	Upper limit
Overall				
Maxillary 1st	80	51.3	43.2	59.3
Maxillary 2nd	54	29.8	23.4	37.1
Age (16–50) (*n* = 205)				
Maxillary 1st	61	61.6	51.3	71.1
Maxillary 2nd	38	35.8	26.9	45.8
Age (>50) (*n* = 132)				
Maxillary 1st	19	33.3	21.7	47.2
Maxillary 2nd	16	21.3	13.0	32.6
Gender (male) (*n* = 129)				
Maxillary 1st	29	47.5	34.8	60.6
Maxillary 2nd	23	33.8	23.1	46.4
Gender (female) (*n* = 208)				
Maxillary 1st	51	53.7	43.2	63.9
Maxillary 2nd	31	27.4	19.7	36.8

The overall distribution of MB-2 canals in maxillary first molars in patients aged 16–50 (61.6%) was substantially >50 years of age (33.3%) (Table 2). There was a statistically significant difference in the MB-2 canal association in the two age groups (*p* < 0.001). Although females (53.7%) had a greater percentage of MB-2 canals in maxillary first molars, it was not significantly higher than males (47.5%) (*p* = 0.454) (Table 2). Among the maxillary second molars, the overall prevalence of MB-2 canals was slightly higher in the 16–50 age group (35.9%) than 50 years of age (21.3%) (*p* = 0.035) (Table 3). There was no significant difference in the association of MB-2 canals in maxillary second molars with respect to gender (*p* = 0.363).

**Table 2:** Association of the presence of MB-2 canals in maxillary first molars by gender, and age groups (*n* = 156).

Variable	*n*	MB-2 canal absent *n* (%)	MB-2 canal present *n* (%)	χ^2^ statistics (*df*)	*P* Value^a^
Age (years)					
16–50	99	38(38.4)	61(61.6)	11.58(1)	<0.001^b^
>50	57	38(66.7)	19(33.3)	11.58(1)	
Gender					
Male	61	32(52.5)	29(47.5)	0.56(1)	0.454
Female	95	44(46.3)	51(53.7)	0.56(1)	

*df* degree of freedom.

^a^Chi-squared test.

^b^Significant difference *(p* < 0.05).

**Table 3:** Association of the presence of MB-2 canals in maxillary second molars by gender, and age groups (*n* = 181).

Variable	*n*	MB-2 canal absent *n* (%)	MB-2 canal present *n* (%)	χ^2^ statistics (*df*)	*P* Value^a^
Age (years)					
16–50	106	68(64.2)	38(35.9)	4.42(1)	0.035^b^
>50	75	59(78.7)	16(21.3)	4.42(1)	
Gender					
Male	68	45(66.2)	23(33.8)	0.83(1)	0.363
Female	113	82(72.6)	31(27.4)	0.83(1)	

*df* degree of freedom.

^a^chi-squared test.

^b^Significant difference (*P* < 0.05).

Among maxillary first molars, the frequency distribution of MB-2 canal conjoins with MB-1 (52.5%, 95% CI: 41.1, 63.7) was slightly greater than MB-2 canals exit via separate foramina (47.5%, 95% CI: 36.3, 58.9) (Table 4). The proportion of single apical foramen in MB-2 canals (51.9%) was also greater than two apical foramina (48.1%) in maxillary second molars. The associations of MB-2 exiting separate apical foramina were significantly greater in ages of 16–50 than age 50 years of age for both maxillary molars (Tables 5 and 6). In the presence of MB-2 canals, the association of MB-2 exit and gender were statistically not significant in both maxillary first (*p* = 0.196) and second molars (*p* = 0.289).

**Table 4:** Prevalence of the number of MB-2 exit(s) for each maxillary molar.

	95% Confidence interval			
	Frequency (*n*)	Percentage (%)	Lower limit	Upper limit
Maxillary First Molars (*n* = 80)				
Single exit	42	52.5	41.1	63.7
Two exits	38	47.5	36.3	58.9
Maxillary Second Molars (*n* = 54)				
Single exit	28	51.9	38.0	65.5
Two exits	26	48.1	34.5	62.0

**Table 5:** Overall association of the different demographic factors with the number of MB-2 exit(s) in maxillary first molars (*n* = 80).

	Single exit *n* (%)	Two exits *n* (%)	Chi-square (*df*)	*P* value^a^
Gender				
Males	18(62.1)	11(37.9)	1.67(1)	0.196
Females	24(47.1)	27(52.9)	1.67(1)	
Age *(years)*				
16–50	29(47.5)	32(52.5)	2.53(2)	0.112
>50	13(68.4)	6(31.6)	2.53(2)	

*df* degree of freedom.

^a^Chi-square test.

Significant difference (*P* < 0.05).

**Table 6:** Overall association of the different demographic factors with the number of MB-2 exit(s) in maxillary second molars (*n* = 54).

	Single exits *n* (%)	Two exits *n* (%)	Chi-square (df)	*P* value^a^
Gender				
Males	10(43.5)	13(56.5)	1.13(1)	0.289
Females	18(58.1)	13(41.9)	1.13(1)	
Age (years)				
16–50	20(52.6)	18(47.4)	0.03(1)	0.860
>50	8(50.0)	8(50.0)	0.03(1)	

*df* degree of freedom.

^a^Chi-square test.

Significant difference (*P* < 0.05).

## Discussion

The present study showed a smaller prevalence of MB-2 canals in the maxillary first and second molars than other similar studies conducted in Asian countries [16, 19, 20, 23]. This could be owing to the relatively small number of CBCT scans (*n* = 263) collected in this study. Several CBCT scans were taken of one quadrant only and potential data from the contralateral quadrant was unavailable. Our study corroborated with a that conducted by Rahman et al. [15] in the Malaysian population. They showed a comparable result of 59.9% of MB-2 canals in maxillary first molars among their population. Similar demographic factors in the Malaysian population, such as Malay ethnicity, may contribute to the comparable prevalence to the present study. Lee et al. [14] and Ratanajirasut et al. [18] conducted studies among the South Korean and Thai populations and reported 28.9% and 29.4% of MB-2 canal distribution in maxillary second molars, respectively. These findings are in agreement with the results of our studies.

There was no significant difference in the frequency distribution of MB-2 canals in both maxillary molars for both genders in the present study. The current finding was consistent with studies conducted by Reis et al. [24], Kewalramani et al. [25] and Al-Habib et al. [26] which revealed that gender did not correlate with the prevalence of MB-2 canals in maxillary molars. In contrast, Kim et al. [13], Lee et al. [14] and Betancourt et al. [12] reported a significantly greater prevalence of MB-2 canals in males than females among their respective demographics and methodologies. The reasoning could be due to females having greater incidence of demineralisation and cortical bone loss [27, 28]. Therefore, the lack of contrast in the radiographs might have impeded the tracing of the MB-2 canals, leading to a lower detection rate in females.

The results (Tables 2 and 3) demonstrated significant differences in the prevalence of MB-2 canals in both maxillary molars within two different age groups. Numerous studies [14, 16, 24, 29–31] conducted had demonstrated a significant correlation of the MB-2 canals where the number of MB-2 canals decreased with increasing age. In the present study, MB-2 canals were more prevalent in those aged below 50 compared to those aged above 50 years old. This can be attributed to the process of aging which leads to a natural progressive reduction in the size of the pulp chamber and calcification of the root canal system [32, 33]. In contrast, Fernandes et al. [30], Faraj [31], and Martins et al. [34] claimed that increasing age did not significantly affect the prevalence of MB-2 canals in the maxillary first and second molars among the Australian, Brazilian, and Kurdistan populations. Differences in ethnicities could be a possible factor in the results’ discrepancies.

In our study, MB-2 canals of maxillary first and second molars shared a common apical foramen with a prevalence of 52.5% and 51.9%, respectively. Those with two separate portals of exit was seen in 47.5% of maxillary first molars and 48.1% of maxillary second molars. It is apparent that more MB-2 canals fuse to the main canal and exit via single apical foramen as compared to exit via two apical foramina in both maxillary molars. The results of our study were in accordance with Ratanajirasut et al. [18], Cleghorn et al. [29], Faraj [31], Martins et al. [34] and Patel & Horner [35] in their respective populations. From the present results, the frequency of two apical exits was observed to have no significant correlation with the age groups. However, a study conducted by Faraj [31] had displayed a more statistically significance in the frequency of two apical foramina in ages between 20 and 50 compared to those aged 50 and above. Fusion of MB-2 canal and MB-1 canal was found more frequently with increasing age due to physiological calcification patterns of the pulp and narrowing of the canals via secondary dentine deposition [32]. This process could account for the significant reduction of two apical foramina in the age group >50 in the present study. However, the results should be interpreted with caution as our sample was small, and the range of ages did not spread extensively in the present study. There was no significant correlation of gender with the number of apical exits among MB-2 canals of both maxillary molars. To our knowledge, only one study was performed by Faraj [31] that investigated and showed non-significant association of gender and the number of MB-2 canal exits. The reasoning remains unclear to which whether gender affects the number of apical portals exits of MB-2 canals.

**Fig. 3:**
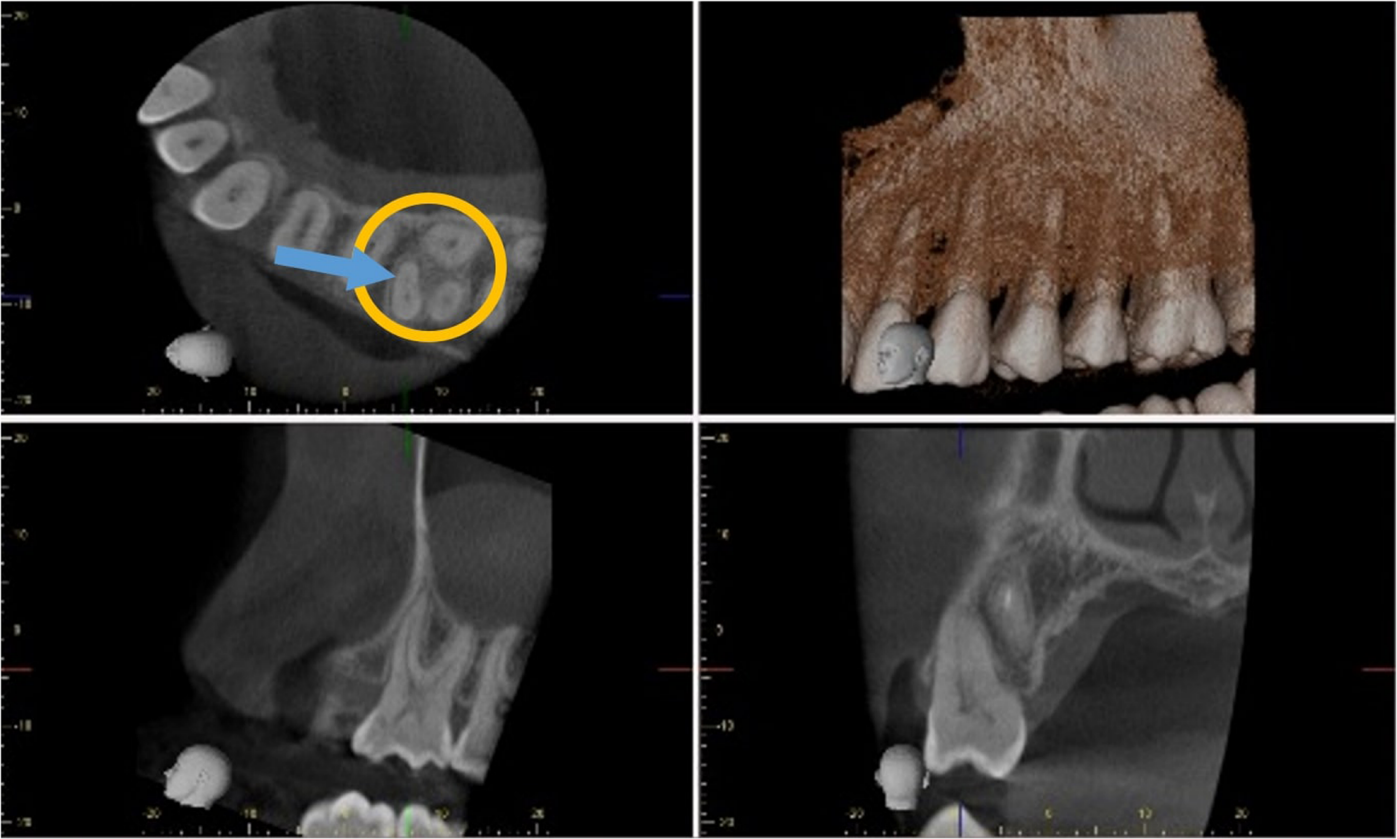
CBCT scan of teeth in the upper left maxillary region. A distinct MB-2 canal (blue arrow) on an axial cross section of an upper first molar (yellow circle).

CBCT may be an efficient tool for detection of MB-2 canals in maxillary molars prior to endodontic treatment. Although it has been reported to have a lower radiation dose than conventional medical tomography [36–38], its use as a routine pre-operative diagnostic tool in endodontics is not currently advocated [39, 40]. As shown in Fig. 3, CBCT allows for precise visualisation of complex root morphology in a three-dimensional manner with views in axial, coronal, and sagittal sections [12, 16]. CBCT scans allow dental practitioners to precisely obtain this valuable information in one single sweep of the machine around the patient’s head. Without the use of CBCT, the same diagnostic information could only be achieved by taking multiple views of two-dimensional radiographs [6, 41]. Consequently, CBCT scan is a highly valuable assessment tool that can potentially aid in location of MB-2 canal and improve endodontic treatment success.

### Limitations

A few limitations have to be addressed that might contribute to the results reported. Out of the 460 CBCT request scans identified, there were scans that could not be retrieved from the database, and had to be inadvertently omitted from the sample. Several scans were requested for one quadrant only negating potential MB-2 canal findings on the contralateral side. Hence, it reduced the number of maxillary molars observed that might hypothetically possess MB-2 canals. Also, the CBCT requests required the referrer to notate the region of interest to be scanned. In 157 of the cases, the region to be scanned were of edentulous areas for implant assessment. This contributed to further loss of sample in the study.

## Conclusion

Identification and effective negotiation of all canals are important determinants for the success of any root canal treatment. Based on our study, the prevalence of MB-2 canals in maxillary first molars and second molars are 51.3% and 29.8% respectively with a higher prevalence for those aged below 50 (61.6% and 35.8% respectively). It is therefore prudent for our clinicians to locate MB-2 canals to achieve a more predictable positive outcome when instituting root canal treatment of maxillary molars amongst the Bruneian population.
